# Characterizing Regional Variability in Lung Cancer Outcomes across Ontario—A Population-Based Analysis

**DOI:** 10.3390/curroncol29120757

**Published:** 2022-12-07

**Authors:** Monica L. Mullin, Jonas Shellenberger, Marlo Whitehead, Michael Brundage, Elizabeth A. Eisenhauer, M. Diane Lougheed, Christopher M. Parker, Geneviève C. Digby

**Affiliations:** 1 Department of Medicine, Queen’s University, Kingston, ON K7L 3N6, Canada; 2 ICES, Queen’s University, Kingston, ON K7L 3N6, Canada; 3 Department of Oncology, Queen’s University, Kingston, ON K7L 3N6, Canada; 4 Division of Respirology, Queen’s University, Kingston, ON K7L 3N6, Canada

**Keywords:** lung cancer, population outcomes, health equity

## Abstract

*Background*: Lung cancer (LC) is the leading cause of cancer-related mortality. In Ontario, Canada, there are significant survival differences for patients with newly diagnosed LC across the 14 provincial regions. *Methods*: A population-based retrospective cohort study using ICES databases from 01/2007–12/2017 identified patients with newly diagnosed LC through the Ontario Cancer Registry and those with LC as the cause of death. Descriptive data included patient, disease, and system characteristics. The primary outcome was 5-year survival by region. *Results*: 178,202 patient records were identified; 101,263 met inclusion criteria. LC incidence varied by region (5.6–14.6/10,000), as did histologic subtype (adenocarcinoma: 27.3–46.1%). Five-year cancer-specific survival was impacted by age, rurality, pathologic subtype, stage at diagnosis, and income quintile. Timely care was inversely related to survival (fastest quintile: HR 3.22, *p* < 0.0001). Adjusted 5-year cancer-specific survival varied across regions (24.1%, HR 1.12; 34.0%, HR 0.89, *p* < 0.001). *Conclusions*: When adjusting for confounders, differences in survival by health region persisted, suggesting a complex interplay between patient, disease, and system factors. A single approach to improving patient care is likely to be ineffective across different systems. Quality improvement initiatives to improve patient outcomes require different approaches amongst health regions to address local disparities in care.

## 1. Introduction

Lung cancer (LC) is the leading cause of cancer-related mortality in Canada [1]. It is the most commonly diagnosed cancer among Canadians [1] and, despite modest improvements in survival over time, the 5-year age-standardized relative survival rate for LC in Canada was only 20.6% as of 2015 [2]. In Canada, health services are organized by province; in Ontario at the time of our review, there were 14 Local Health Integrated Network regions (LHINs). While Ontario has the highest provincial average 5-year relative LC survival at 24.4% [3], data from 2009–2013 demonstrate significant variability in 5-year relative survival across LHINs [4], ranging from 15.0% to 26.1% [5]. While substantial provincial variability in 5-year relative survival also exists for other cancer types [5], it is most pronounced for LC.

Several international studies have demonstrated treatment and survival disparities in the LC patient population with rural populations having higher LC incidence, a higher proportion of late-stage disease, lower odds of receiving treatments, and increased mortality compared to urban populations [6,7,8,9]. This is postulated to be due to higher rural smoking rates, socioeconomic deprivation, and differences in access to diagnostic or treatment facilities [10,11]. Other studies have not found any association between rurality and delays in care, which may be due to the “sicker quicker” effect; if patients in rural populations present with later-stage disease, their care may be expedited [12,13]. These potential inequities are of concern for a province such as Ontario, which has a large geographic footprint, a significant proportion of rural communities, and variability in socioeconomic status.

While patient and disease factors, such as age, comorbidities, and pathologic subtype of LC affect LC outcomes [14,15,16,17], systemic barriers in access to care, including coordination of care, distances to a cancer center or specialist clinics may also contribute to regional care inequities [18,19,20]. In order to fulsomely address the barriers that create LC inequities, a detailed understanding of care differences that contribute to this systemic problem is required, such that multilevel system changes can be implemented to achieve desired cancer care outcomes [21].

We conducted a detailed, population-based analysis to better understand the variability in LC outcomes across Ontario and define patient, disease, and system factors that contribute to regional differences. In doing so, we aimed to identify factors amenable to modification in future initiatives to address these outcome variations.

## 2. Study Design and Methods

We conducted a population-based retrospective cohort study (1 January 2007 to 31 December 2017) using data obtained from universal healthcare coverage in Ontario, Canada housed at ICES (formerly known as the Institute for Clinical Evaluative Sciences). ICES is a non-profit, independent research institute that collects and analyzes healthcare data under Ontario’s health information privacy law, with a mandate to improve health and healthcare. The primary databases used in this project are shown in Table 1. These datasets were linked using unique encoded identifiers and analyzed at ICES. This study was approved by the Queen’s University Human Research Ethics board. STROBE reporting guidelines were used (Appendix A) [22].

### 2.1. Case Definitions, Demographics, and Study Outcomes

Cases were defined as patients over 18 years of age with a new diagnosis of LC between 1 January 2007 and 31 December 2017, by topography coding consistent with LC in OCR (C34) and exclusion of pathology not consistent with LC (e.g., hematopoietic malignancies, melanoma, and Kaposi sarcoma), which is in keeping with Cancer Care Ontario’s definition. Additional patients were identified if LC was listed as a cause or comorbidity on the death certificate, if not already captured in OCR data. The maximum follow-up date was 31 December 2019, at death, or end of OHIP eligibility. A lookback window was defined as 2 years prior to the diagnosis date. Data were collected and analyzed by LHIN, including patient characteristics (age, sex, income quintile defined as nearest neighborhood income quartile based on postal code, rurality index, type of nearest hospital, and co-morbidities) and disease characteristics (pathologic subtype and stage at diagnosis). 

Our primary outcomes were defined as all-cause survival and cancer-related survival calculated by LHIN. Exclusion criteria included: diagnosis outside the study period, an invalid number in the ICES database, prior diagnosis of LC (i.e., second primary LC), age < 18 years at the time of diagnosis, invalid date of death, not a resident of Ontario during the diagnostic phase, or no associated LHIN. 

### 2.2. Statistical Approach

We used a Cox-model approach to compare overall and cancer-specific 5-year survival for LC across LHINs, adjusted for patient age, sex, income quintile, rurality index, distance to the nearest hospital (in kilometers), nearest hospital type (academic, community, small, unknown), LC stage at diagnosis, LC histology, co-morbidity status by ACG, timeliness of care (divided into 5 quintiles), and assessment by medical or radiation oncology. For this analysis, the timeliness of treatment, radiation, and medical oncology visits were modeled as time-varying covariates. The Wald Chi-Square test was used to assess for statistical differences between groups. Patients diagnosed at death were excluded from the survival analysis. Statistical analyses were performed using SAS version 9.4 (SAS Institute, Inc., Cary, NC, USA). The Johns Hopkins ACG® system version 10 was used to compute ACGs.

## 3. Results

**Figure 1:**
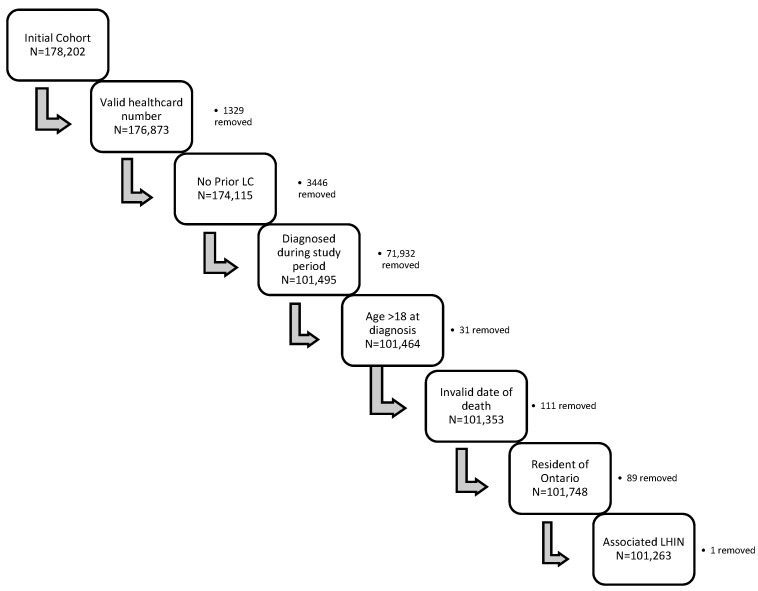
Study Cohort inclusion and exclusion criteria. This figure is a graphical representation of the flow of patient exclusion for our study, including the exclusion criteria, the total number of patients at each step (N), and the number of patients removed for each criterion.

Our cohort identified 178,202 patient records of which 101,263 patients met the inclusion criteria (Figure 1). Within our cohort, most patients were identified by date of diagnosis (*n* = 97,330) while the remaining (*n* = 3933) were identified by death records alone. 

### 3.1. Patient Characteristics

Patient characteristics of the entire cohort are summarized in Table 2. The median age of LC diagnosis was 71 years (IQR 63–79) and 48.3% were female. The majority of patients lived in an urban area (61.9%) and resided closest to a community hospital (68.4%). Median number of ACGs in the 2 years preceding diagnosis was 8 (IQR 6–11). The most common LC histologic subtype was adenocarcinoma (35.2%), closely followed by poorly differentiated carcinoma (33.5%). Stage IV disease was the most common stage at presentation (41.9%). Patients identified at death alone were older with a median age of 79 years (IQR 71–85) and had more comorbidities compared to the rest of the cohort (72.5% with >3 major ACGs vs. 35.9% in patients with OCR diagnosis date). 

These patient characteristics varied across LHINs, as shown in Table 3. Median age at diagnosis was similar, ranging from 71 to 73 years. Median rurality index varied from 0 to 34 between regions. Significant variability in LC patient income quintiles was identified between LHINs with the proportion of patients in the lowest income quintile ranging from 13.0% to 30.7% between regions. There was also significant variability in the closest hospital type, with the percentage of patients living closest to a teaching hospital ranging from 0% in six LHINs to 59.7% in one LHIN. Meanwhile, LC incidence also varied across LHINs, ranging from 5.6/10,000 in Central West (LHIN 5) to 14.6/10,000 in North East (LHIN 13).

### 3.2. Lung Cancer Disease Characteristics

**Figure 2:**
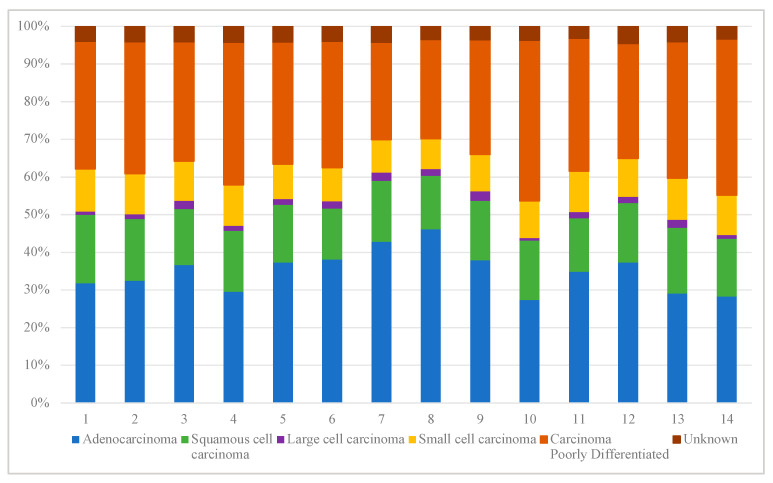
Proportion of histologic subtypes of lung cancer, by LHIN. This graph demonstrates the breakdown of pathologic subtype for patients diagnosed with LC in each LHIN throughout the study period. There are differences between LHINs, with some (LHIN 5–8) having higher proportions of more favorable pathology, such as adenocarcinoma with others (LHIN4, 10, 13–14) having higher proportions of less favorable pathology such as small cell, poorly differentiated and unknown.

**Figure 3:**
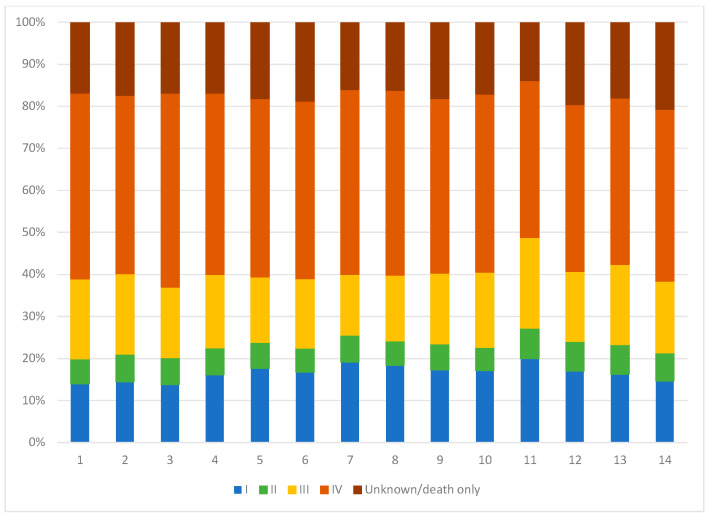
Stage of disease at lung cancer diagnosis, by LHIN. This graph demonstrates the stage of LC at diagnosis for patients in each LHIN throughout the study period. There are differences between LHINs, with some having higher proportions of early stage (LHIN 5–8, 11) and others having more patients with stage unknown or stage IV (LHIN 3, 12, 14).

There was variability in LC disease characteristics across LHINs. The percent of patients with adenocarcinoma histologic subtype varied, with South East (LHIN 10) having the lowest proportion of patients with this subtype (27.3%) and Central (LHIN 8) having the highest proportion (46.1%). Meanwhile, the proportion of patients with poorly differentiated carcinomas not otherwise specified (NOS) ranged from 42.6% in the South East (LHIN 10) to 25.8% in Toronto Central (LHIN 7) (Figure 2). Evaluation of LC staging breakdown by LHIN demonstrated minimal variation but with an overall high proportion of patients presenting with advanced-stage disease across all LHINs (Figure 3).

### 3.3. Lung Cancer Outcomes

#### 3.3.1. Patient and Disease Characteristics

Several patient- and disease-related covariates were found to significantly impact the adjusted 5-year cancer-specific survival (Table 3). Older age was associated with a higher hazard ratio (HR) for death (HR 1.03, *p* < 0.0001). The risk of death was found to be inversely proportional to the income quintile. Patients living in a small urban region had a modest but significantly higher risk of death (HR 1.05, *p* < 0.0001) compared with those living in a large urban region. Survival was modestly impacted if the nearest hospital was a small hospital, as opposed to a teaching or community hospital (HR 1.04, *p* = 0.0480), but the distance a patient lived from a hospital had no impact. LC stage at diagnosis, histologic subtype, and patient comorbidities all had higher HR for cancer-specific mortality compared to other factors such as type of nearest hospital, income quintile, and rurality index (Table 4).

#### 3.3.2. System Factors 

LC survival varied between healthcare regions, with the all-cause 5–year survival ranging from 14.7% in LHIN 3-Waterloo Wellington (unadjusted HR 1.17, *p* < 0.0001 and adjusted HR 1.09, *p* < 0.0001, compared with reference LHIN) to 22.1% in LHIN 8-Central (unadjusted HR 0.92, *p* < 0.0001 and adjusted HR 0.88, *p* < 0.0001, compared with reference LHIN).

The 5-year cancer-specific survival also varied across LHINs, ranging from 24.1% in LHIN 3-Waterloo Wellington (unadjusted HR 1.20, *p* < 0.001 and adjusted HR 1.12, *p* < 0.0001, compared with reference LHIN) to 34.0% in LHIN 8-Central (unadjusted HR 0.91, *p* < 0.0001 and adjusted HR 0.89, *p* < 0.0001, compared with reference LHIN) (Table 5). 

#### 3.3.3. Other System Factors 

**Figure 4:**
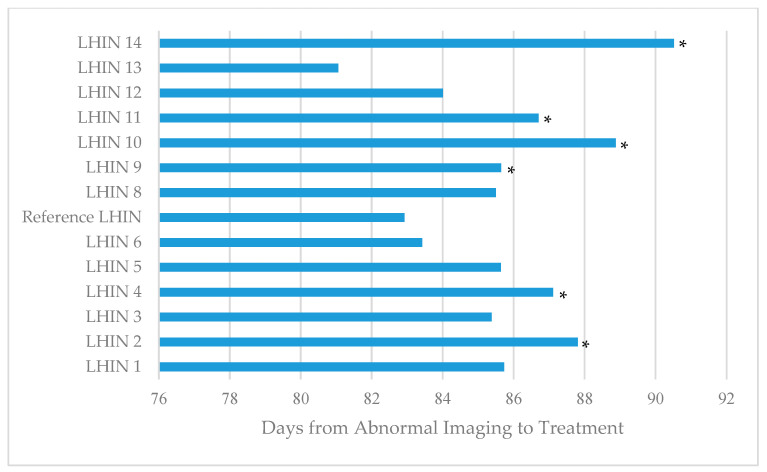
Timeliness of care from first abnormal imaging to LC treatment. This graph shows the average number of days from the first abnormal imaging to the first treatment (including radiation, surgery, or systemic therapy) for lung cancer across LHINs. *denotes significance by pairwise *t*-tests for each of the LHINs compared to LHIN 7, using Šidák correction for multiple comparisons, *p* < 0.05.

Timeliness of care varied across LHINs, with time from first abnormal imaging to treatment ranging from 81.1 days in LHIN 13-North East to 90.5 days in LHIN 14-North West (Figure 4), but faster care did not improve survival, with patients receiving faster care having an increased risk of death (HR 3.22 for shortest quintile compared with the longest quintile, *p* < 0.0001).

There was also variation amongst LHINs with respect to the number of radiation oncology (RO) and medical oncology (MO) visits prior to treatment initiation, or in the 90 days after diagnosis if the patient did not receive treatment. This ranged from an average of 0.42 RO visits/patient in LHIN 4-Hamilton Niagara to 0.71 RO visits/patient in LHIN 10-South East. The visits with MO also varied from 0.17 visits/patient in LHIN 4-Hamilton Niagara to 0.5 visits/patient in LHIN 5-Central West. Assessment by RO was associated with worse survival (HR 1.28, *p* < 0.0001), while assessment by MO was associated with improved survival (HR 0.94, *p* < 0.0001) (Table 3). 

**Figure 5:**
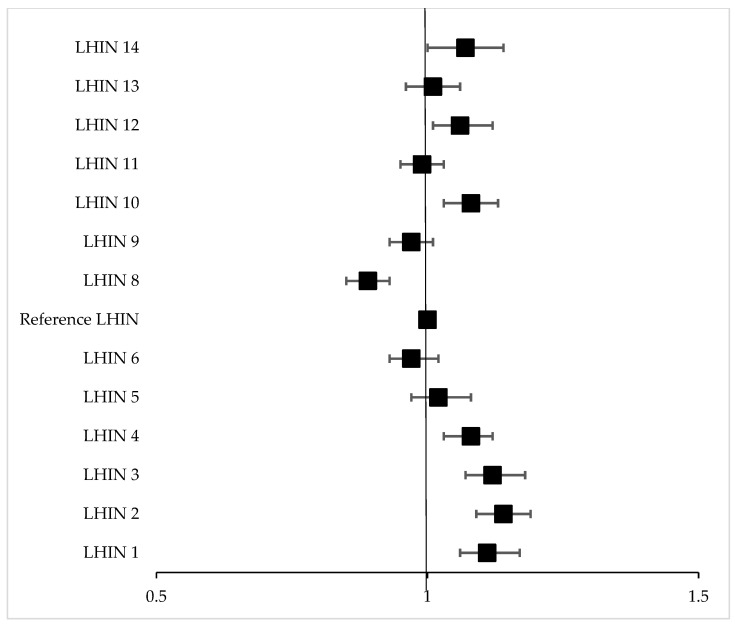
Adjusted hazard ratios for cancer-specific mortality by LHIN. This graph shows the HRs for cancer-specific mortality calculated by Cox modeling and adjusting for patient age, sex, income quintile, rurality index, distance to the nearest hospital, stage, histology, co-morbidity status, timeliness of care, and access to specialty assessment. After adjusting for these variables, the performance of some LHINs improved, and while there was less disparity amongst LHINs, significant differences remained.

LC survival and HR estimates varied between the unadjusted and the adjusted analyses (Table 4); after adjusting for patient age, sex, income quintile, rurality index, distance to the nearest hospital, stage, histology, co-morbidity status, timeliness of care, and specialist assessment, there was less variability across LHIN and improvement in the survival estimate for several LHIN in the adjusted analysis. Even still, there remained a significant difference in survival in the adjusted analysis for several LHINs compared with the reference LHIN (Figure 5).

## 4. Discussion

Significant variability in LC survival exists across health regions in Ontario. Our data demonstrate that both patient- and disease-related characteristics contribute significantly to this observed variability in survival, with the LC stage at diagnosis and histologic subtype exhibiting the greatest impact on survival. However, variability in patient and disease characteristics between health regions did not account for all the observed variability in adjusted cancer-specific survival across LHINs, suggesting that other system factors may play a contributing role. We adjusted for several system factors in a multivariate analysis including timeliness of care, size of the closest hospital, and specialist consultation, yet variability in survival across health regions persisted. 

As clinically anticipated, disease characteristics had the largest impact on the risk of mortality, with the highest HRs for mortality attributed to the increasing stage of the disease. Histologic subtypes also contributed significantly to survival, with non-adenocarcinoma subtypes associated with increased HR for death. This is supported by research illustrating that stage of the disease is directly related to survival and certain histologic subtypes are associated with worse outcomes [15,16,17]. Most notably, patients who declined or were unable to proceed with staging, represented as an unknown stage, had a significantly increased risk of death (HR = 6.29, *p* < 0.0001), which may relate to the severity of illness at diagnosis or willingness or wellness to undergo treatment. 

This study is in keeping with prior knowledge that cancer-associated survival differences exist across Ontario and that patient characteristics play a contributing role in survival outcomes. A previous study demonstrated that cancer-specific 5-year survival varied from 52% to 72% at different hospitals across Ontario and these differences were present across many cancer types [23]. However, adjusting for socioeconomic and urban-rural status minimally reduced variation, suggesting that other factors likely contributed to this variability. Other studies have found a correlation between LC outcomes and income, with patients of lower income being nearly twice as likely to be diagnosed with LC, and more likely to have advanced disease. Even when presenting with earlier stages of the disease, lower-income patients are less likely to receive curative treatments [11]. Canadian data suggest that LC incidence is inversely correlated with income and geography, with those of lower income being twice as likely to be diagnosed with LC. However, LC survival is more closely correlated with income and less so with geography in Canada [24]. Similar findings have been found internationally, with a study in Germany demonstrating lower survival in lower-income groups regardless of location [11]. As well, a UK study found a relationship between low-income quintiles, later stage at diagnosis, and worse survival [7].

Although these disease factors may appear to relate to unmodifiable characteristics, there may be more complex relationships between histologic subtypes and late presentations of disease that still relate to health regions and the structure of care systems. For example, smoking rates were unable to be included in this study but are associated with squamous cell carcinoma, carcinoma NOS, and small cell carcinoma, which have a worse prognosis when compared to adenocarcinoma. Smoking rates vary across Ontario from as low as 7.6% to 26.2% [25], which may impact the noted differences in histology amongst LHINs. Furthermore, there is a complex inter-relatedness between smoking and other factors that are associated with health inequity, such as immigrant status, aboriginal ancestry, and substance use, which may in turn impact cancer risk and survival outcomes. While efforts such as smoking cessation or early LC diagnosis with screening programs may be impactful in improving overall cancer outcomes, the implementation of such efforts remains a challenge due to the complex associations with other barriers to care delivery and interconnectedness with the aforementioned factors associated with health inequities. Meanwhile, those with pathology reported as carcinoma not otherwise specified had an increased risk of mortality (HR = 1.71, *p* < 0.0001), which could be explained by the aggressive nature of this histologic subtype and/or the limited therapeutic options. With advances in tumor markers, it is recommended that carcinoma NOS be used to report less than 5% of cases, yet we found that all LHINs exceeded this target, with many >30% [26].

The concept of “sicker quicker” care is also well documented in the literature and can explain the observed higher mortality despite faster care for many patients [12]. Given this complex relationship between timely care and patient outcomes, it is hard to identify the benefits of timely care across LC patients. However, delays in care are clearly undesirable and cannot improve survival rates. In our study, the time from first abnormal imaging to diagnosis was approximately 80–90 days, when the literature would support a shorter interval of fewer than 90 days from symptom onset through to treatment [27]. As such, this is an area that could likely be improved across the province. Similarly, the apparent impact of radiation oncology assessment being associated with lower survival rates is likely due to associations with other patient factors. Often, patients who are too frail to receive other treatments, such as surgery or chemotherapy, are offered palliative radiation treatments, which may not have occurred at the primary tumor site. We were unable to differentiate the location of radiation therapy and perhaps this association may not have been seen if the analysis was limited to radiation of the primary tumor. It is also known in the literature that treatment with systemic therapy is associated with improved survival [28] and thus, it follows that assessment by medical oncology would offer this benefit.

In terms of possible additional contributing factors, there are many complexities in the medical system which it is difficult to account for with database analysis. For example, while we did not show improved survival with more timely care, we are likely not capturing all contacts with the healthcare system that exists along the continuum of patient care As well, patient care that included multidisciplinary discussion or care within diagnostic assessment pathway models could not be captured but has been shown to improve cancer-specific survival [29,30]. The interplay amongst these multiple layers of the healthcare system and transitions of care is an essential component of quality improvement research [21]. Studying the care systems in areas with the best survival may lead to improvement in survival across regions.

One limitation of our study was the inability to obtain LHIN-level smoking rates or patient access to smoking cessation counseling. However, wide variability in smoking rates [25] may also impact the survival outcomes. Given the findings of this study suggesting that histologic subtype significantly impacts survival, further studies outlining smoking incidence in each LHIN may be beneficial. Another limitation is related to the imperfect nature of database cohort analyses, recognizing that the coding of information is not always accurate. Our cohort definition was in keeping with Cancer Care Ontario’s definition, in an effort to minimize inaccuracies.

### Learning Opportunities to Guide Provincial Quality Improvement Efforts in Lung Cancer Care

Overall, the observed variability in LC survival outcomes as well as the significant variability in patient, disease, and system factors across health regions in Ontario suggests that there is no single strategy that will improve LC outcomes across the province. Rather, the findings from this study highlight the need for regional quality improvement strategies that target the multitude of specific barriers faced by individual health regions. As underscored in the literature to achieve healthcare delivery that satisfies all domains of healthcare quality, change is required at multiple levels of the system. Ignoring the complexity of the multilevel environment in which care occurs does not achieve the desired improvements in care. Improvement efforts will need to be situated within a framework such as that suggested by Taplin et al that recognizes the need to consider care as a process in a dynamic system and the need to influence multiple levels of the system to achieve an improved quality of cancer care and improved cancer-related health outcomes [21].

While many of the factors associated with LC survival are not modifiable, such as age and comorbidities, there are still some patient, disease, and system characteristics for which interventions could impact outcomes. Even still, variability in patient and disease characteristics across health regions did not account for all the variability seen in LC survival outcomes, indicating that system factors play a contributing role in survival, beyond timeliness of care, closest hospital, or access to specialty assessment. As such, quality improvement efforts will need to consider the multiple unique system barriers affecting survival in each health region. A framework, such as that by Taplin et al, can help in the design of effective and targeted improvement strategies that address multilevel influences on the cancer care continuum [21].

Additionally, while timeliness of care has historically been tracked as a priority metric by governing bodies such as Cancer Care Ontario, it is not possible in this research to show an impact of timeliness despite significant observed variability in the timeliness of care across health regions, presumably due to the “sicker quicker” effect. There remain opportunities to improve the timeliness of care across the entire province, but the strategies taken to enact these system changes will vary significantly across health regions due to the differences in the populations served. Quality metrics with clear relevance to LC survival should be monitored and could include process measures such as the percentage of patients diagnosed with early-stage disease, equitability of access to care for patients of lower income quintiles, and percentage of patients receiving LC treatment. Further evaluation as to the impact of other regional system characteristics, such as access to diagnostic assessment programs, specialist assessment, and health resource utilization is needed to further define modifiable system factors that contribute to LC survival outcomes. Finally, since there is no reason to believe that these variations across regions are unique to the province of Ontario, our findings are likely relevant to other jurisdictions with similar healthcare systems in Canada and elsewhere.

## 5. Conclusions

Significant variability in LC survival exists across health regions in Ontario, not completely accounted for by variability in those patients and disease characteristics available in our data, suggesting that other patients or system factors play a contributing role. A regional approach to improvement efforts will be required to identify the root causes contributing to unique regional system factors that influence survival to develop targeted improvement strategies. Governing bodies should consider the uniqueness of individual health regions to ensure that resources are equitably distributed in such a way that addresses unique regional needs and should consider tracking additional quality metrics, beyond timeliness of care, that have the most potential to impact LC survival.

## Figures and Tables

**Table 1:** ICES databases used for cohort creation and data analysis.

Acronym	Database Name	Content
OHIP	Ontario Health Insurance Plan	Physician Claims Database which records all claims made by physicians for insured services
CIHI DAD	Canadian Institute for Health Information Discharge Abstract Database	Diagnostic and procedural information from hospitalizations
NACRS	National Ambulatory Care Reporting System	Ambulatory procedural information
SDS	Same Day Surgery Database	Procedural information for patients without an associated hospital stay
IPDB	ICES Physician Database	Information on physician specialty and location of practice
RPDB	Registered Persons Database	Demographic and vital status information
PCCF	Postal Code Conversion File	Provides geographic location of patients and services
INST	Institution Information System	Information about Ontario Healthcare institutions
OCR	Ontario Cancer Registry	Cancer-related characteristics including diagnosis date, pathologic subtype, and treatment information
ALR	Cancer Activity Level Reporting	Cancer-related characteristics including diagnosis date, pathologic subtype, and treatment information
ORDG	Office of the Registrar General—Deaths	Vital statistics and death information
ACG Algorithm	Adjusted Clinical Groups	Developed by Johns Hopkins University, used to provide information on comorbidity status

**Table 2:** Patient characteristics for entire lung cancer cohort.

VARIABLE	VALUE	OCR Diagnosis Data	Diagnosis at Death	TOTAL
Cohort Size		*N* = 97,330	*N* = 3933	*N* = 101,263
Age at diagnosis	Median (IQR)	71 (63–79)	79 (71–85)	71 (63–79)
Female		47,157 (48.45%)	1743 (44.51%)	48,896 (48.30%)
2008 Rurality Index for Ontario	Median (IQR)	4 (0–24)	5 (0–24)	4 (0–24)
	Missing	1289 (1.32%)	58 (1.48%)	1347 (1.33%)
	Large Urban	60,270 (61.92%)	2375 (60.42%)	62,645 (61.87%)
	Small Urban	25,835 (26.54%)	1068 (27.15%)	26,903 (26.57%)
	Rural	9936 (10.21%)	432 (10.96%)	10,368 (10.24%)
Sum of ADGs in 2 years preceding dx	Median (IQR)	8 (6–11)	11 (8–13)	8 (6–11)
Sum of major ACGs in 2 years preceding dx	0	7402 (7.61%)	85 (2.16%)	7487 (7.39%)
	1	25,023 (25.71%)	259 (6.59%)	25,282 (24.97%)
	2	29,984 (30.81%)	736 (18.71%)	30,720 (30.34%)
	3	19,697 (20.24%)	1044 (26.54%)	20,741 (20.48%)
	4+	15,224 (15.64%)	1809 (46.00%)	17,033 (16.82%)
Nearest Census-Based Neighbourhood Income Quintile	Missing	362 (0.37%)	10 (0.25%)	372 (0.37%)
	1 (lowest)	23,562 (24.21%)	968 (24.61%)	24,530 (24.22%)
	2	21,636 (22.23%)	864 (21.97%)	22,500 (22.22%)
	3	18,885 (19.40%)	776 (19.73%)	19,661 (19.42%)
	4	17,449 (17.93%)	671 (17.06%)	18,120 (17.89%)
	5 (highest)	15,436 (15.86%)	644 (16.37%)	16,080 (15.88%)
LHIN	01. Erie St. Clair	5957 (6.12%)	247 (6.28%)	6204 (6.13%)
	02. South West	7926 (8.14%)	338 (8.59%)	8264 (8.16%)
	03. Waterloo Wellington	4668 (4.80%)	198 (5.03%)	4866 (4.81%)
	04. Hamilton Niagara Halton	2727 (13.08%)	562 (14.29%)	13,289 (13.12%)
	05. Central West	3684 (3.79%)	163 (4.14%)	3847 (3.80%)
	06. Mississauga	5520 (5.67%)	227 (5.77%)	5747 (5.68%)
	07. Toronto Central	6822 (7.01%)	302 (7.68%)	7124 (7.04%)
	08. Central	8886 (9.13%)	323 (8.21%)	9209 (9.09%)
	09. Central East	11,791 (12.11%)	445 (11.31%)	12,236 (12.08%)
	10. South East	5519 (5.67%)	209 (5.31%)	5730 (5.66%)
	11. Champlain	10,474 (10.76%)	345 (8.77%)	10,819 (10.68%)
	12. North Simcoe Muskoka	4356 (4.48%)	209 (5.31%)	4565 (4.51%)
	13. North East	6767 (6.95%)	285 (7.25%)	7052 (6.96%)
	14. North West	2232 (2.29%)	78 (1.98%)	2311 (2.28%)
Histologic subtype	No Tissue	0 (0.00%)	3933 (100%)	3933 (3.88%)
	Adenocarcinoma	35,661 (36.64%)		35,661 (35.22%)
	Poorly Differentiated	33,907 (34.84%)		33,907 (33.48%)
	Large cell	1740 (1.79%)		1740 (1.72%)
	Small cell	10,176 (10.46%)		10,176 (10.05%)
	Squamous cell	15,846 (16.28%)		15,846 (15.65%)
Stage at Diagnosis	Unknown	13,507 (13.88%)	3933 (100%)	17,440 (17.22%)
	I	17,113 (17.58%)		17,113 (16.90%)
	II	6458 (6.64%)		6458 (6.38%)
	III	17,788 (18.28%)		17,788 (17.57%)
	IV	42,464 (43.63%)		42,464 (41.93%)

**Table 3:** Patient characteristics stratified by LHIN.

LHIN	Median Age at Diagnosis (IQR)	Nearest Census-Based Neighbourhood Income Quintile					Median Rurality Index (IQR)	Median ADGs Sum (IQR)	Incidence /10,000
		1	2	3	4	5			
1	71 (63–78)	1534 (24.83%)	1440 (23.30%)	1206 (19.51%)	1098 (17.76%)	877 (14.19%)	11 (0–18)	8 (6–11)	11.78
2	71 (64–79)	1947 (23.65%)	1871 (22.72%)	1679 (20.39%)	1435 (17.42%)	1244 (15.10%)	23 (0–40)	8 (6–10)	10.78
3	71 (63–79)	1256 (25.84%)	1219 (25.08%)	855 (17.59%)	773 (15.90%)	748 (15.39%)	5 (4–7)	7 (5–10)	8.20
4	72 (64–79)	3385 (25.52%)	3175 (23.93%)	2533 (19.09%)	2222 (16.75%)	1891 (14.25%)	3 (0–8)	8 (6–11)	11.50
5	71 (62–78)	674 (17.54%)	970 (25.25%)	1069 (27.82%)	619 (16.11%)	507 (13.20%)	2 (2–6)	9 (6–11)	5.64
6	72 (63–80)	744 (12.95%)	921 (16.04%)	1206 (21.00%)	1485 (25.86%)	1384 (24.10%)	0 (0–2)	9 (6–11)	6.16
7	72 (63–80)	2128 (29.93%)	1435 (20.19%)	971 (13.66%)	893 (12.56%)	1633 (22.97%)	0 (0–0)	9 (6–12)	6.74
8	73 (64–80)	1725 (18.76%)	1885 (20.50%)	1740 (18.93%)	2157 (23.46%)	1669 (18.15%)	2 (0–6)	9 (7–11)	6.31
9	72 (63–79)	3351 (27.41%)	3061 (25.04%)	2355 (19.27%)	1859 (15.21%)	1574 (12.88%)	5 (0–28)	8 (6–11)	9.61
10	71 (63–78)	1665 (29.09%)	1307 (22.83%)	1072 (18.73%)	960 (16.77%)	694 (12.12%)	24 (6–36)	8 (6–10)	14.07
11	71 (63–78)	2335 (21.61%)	2301 (21.29%)	2218 (20.52%)	2147 (19.87%)	1795 (16.61%)	0 (0–35)	8 (6–11)	10.34
12	71 (63–79)	1052 (23.10%)	783 (17.19%)	921 (20.22%)	910 (19.98%)	856 (18.80%)	34 (17–43)	8 (6–10)	12.30
13	71 (64–78)	2164 (30.73%)	1643 (23.33%)	1290 (18.32%)	1100 (15.62%)	807 (11.46%)	24 (3–66)	8 (6–10)	14.69
14	71 (63–79)	523 (22.66%)	457 (19.80%)	519 (22.49%)	427 (18.50%)	370 (16.03%)	0 (0–80)	8 (5–10)	11.67

**Table 4:** Multivariable Cox modeling demonstrating the impact of patient, disease, and system characteristics on cancer-specific mortality for all LC patients in Ontario.

Variable	Value	HR (95% DI)	*p*-Value
Age		**1.03 (1.03–1.03)**	**<0.0001**
Neighborhood Income Quintile	1—Lowest quintile	Reference	
	2	**1.22 (1.19–1.25)**	**<0.0001**
	3	**1.14 (1.1–1.18)**	**<0.0001**
	4	**1.09 (1.06–1.12)**	**<0.0001**
	5—Highest quintile	**1.08 (1.05–1.11)**	**<0.0001**
Rurality Index	Large Urban	Reference	
	Small Urban	**1.05 (1.03–1.08)**	**<0.0001**
	Rural	1.01 (0.97–1.05)	0.6202
	Missing	1.05 (0.97–1.13)	0.2685
Hospital Type	Teaching	Reference	
	Community	1.02 (0.99–1.05)	0.1470
	Small	**1.04 (1.00–1.09)**	**0.0480**
	Missing	**1.05 (1.01–1.09)**	**0.0260**
Distance to the closest hospital		1.00(1.00–1.00)	0.2635
Stage at diagnosis	I	Reference	
	II	**2.00 (1.91–2.11)**	**<0.0001**
	III	**3.75 (3.61–3.89)**	**<0.0001**
	IV	**7.13 (6.88–7.39)**	**<0.0001**
	Missing	**6.29 (6.05–6.54)**	**<0.0001**
Histological subtype	Adenocarcinoma	Reference	
	Poorly Differentiated/NOS	**1.71 (1.67–1.74)**	**<0.0001**
	Large cell carcinoma	**1.29 (1.21–1.37)**	**<0.0001**
	Small cell carcinoma	**1.20 (1.16–1.23)**	**<0.0001**
	Squamous cell carcinoma	**1.16 (1.14–1.20)**	**<0.0001**
Sum of major ADGs in 2 years preceding dx	0	Reference	
	1	0.99 (0.96–1.03)	0.6741
	2	1.02 (0.99–1.06)	0.1840
	3	**1.08 (1.05–1.12)**	**<0.0001**
	4+	**1.23 (1.18–1.27)**	**<0.0001**
Timeliness from first abnormal imaging to treatment	1—Fastest quintile	Reference	
	2	**3.22 (3.13–3.32)**	**<0.0001**
	3	**2.17 (2.11–2.23)**	**<0.0001**
	4	**1.71 (1.66–1.76)**	**<0.0001**
	5—Slowest quintile	**1.59 (1.54–1.63)**	**<0.0001**
Assessed by Radiation Oncology	No	Reference	
	Yes	**1.28 (1.26–1.31)**	**<0.0001**
Assessed by Medical Oncology	No	Reference	
	Yes	**0.94 (0.92–0.96)**	**<0.0001**

Bold values denote statistical significance by Wald-Chi squared test, with *p* < 0.05.

**Table 5:** 5-year survival and hazard ratios for cancer-specific mortality for all patients by LHIN.

	5-Year Survival (95% CI)		Unadjusted Model		Adjusted Model	
LHIN	All-Cause Survival	Cancer-Specific Survival	HR (95% CI)	*p*-Value	HR (95% CI)	*p*-Value
1	0.150 (0.140–0.160)	**0.252 (0.239–0.266)**	**1.20 (1.15–1.26)**	**<0.0001**	**1.11 (1.06–1.17)**	**<0.0001**
2	0.157 (0.148–0.166)	**0.269 (0.257–0.281)**	**1.17 (1.12–1.22)**	**<0.0001**	**1.14 (1.09–1.19)**	**<0.0001**
3	0.147 (0.136–0.159)	**0.241 (0.226–0.256)**	**1.20 (1.14–1.25)**	**<0.0001**	**1.12 (1.09–1.19)**	**<0.0001**
4	0.163 (0.156–0.170)	**0.285 (0.275–0.295)**	**1.11 (1.07–1.15)**	**<0.0001**	**1.08 (1.03–1.12)**	**0.0004**
5	0.200 (0.186–0.215)	0.317 (0.299–0.335)	1.01 (0.96–1.07)	0.6718	1.02 (0.97–1.08)	0.4271
6	0.202 (0.191–0.214)	0.312 (0.297–0.326)	1.01 (0.97–1.06)	0.6067	0.97 (0.93–1.02)	0.2307
7	0.199 (0.189–0.210)	0.320 (0.306–0.334)	Reference		Reference	
8	0.221 (0.212–0.231)	**0.340 (0.328–0.353)**	**0.91 (0.88–0.95)**	**<0.0001**	**0.89 (0.85–0.93)**	**<0.0001**
9	0.192 (0.185–0.200)	0.318 (0.308–0.328)	1.00 (0.96–1.04)	0.9318	0.97 (0.93–1.01)	0.1519
10	0.147 (0.136–0.157)	**0.253 (0.239–0.268)**	**1.19 (1.14–1.25)**	**<0.0001**	**1.08 (1.03–1.13)**	**0.0031**
11	0.190 (0.182–0.199)	0.312 (0.301–0.323)	0.99 (0.95–1.03)	0.6943	0.99 (0.95–1.03)	0.6458
12	0.187 (0.175–0.200)	**0.309 (0.292–0.326)**	1.04 (0.99–1.09)	0.1286	**1.06 (1.01–1.12)**	**0.0302**
13	0.152 (0.143–0.162)	**0.277 (0.264–0.291)**	**1.10 (1.05–1.15)**	**<0.0001**	1.01 (0.96–1.06)	0.8073
14	0.153 (0.137–0.170)	**0.266 (0.243–0.289)**	**1.14 (1.08–1.22)**	**<0.0001**	**1.07 (1.00–1.14)**	**0.0440**

Bold values denote statistical significance by Wald-Chi squared test, with *p* < 0.05.

## Data Availability

The datasets generated during and/or analyzed during the current study are not publicly available but are available from the corresponding author on reasonable request. Please note in keeping with ICES privacy policy, data including N < 5 will not be released due to risk of re-identification.

## Appendix A

**Table A1:** STROBE statement—checklist of items that should be included in reports of observational studies.

	Item No.	Recommendation	Page No.	Relevant Text from Manuscript
**Title and abstract**	1	(*a*) Indicate the study’s design with a commonly used term in the title or the abstract	1	*A population-based retrospective cohort study*
		(*b*) Provide in the abstract an informative and balanced summary of what was done and what was found	1	
**Introduction**				
Background/rationale	2	Explain the scientific background and rationale for the investigation being reported	1-2	
Objectives	3	State specific objectives, including any prespecified hypotheses	2	*We conducted a detailed, population-based analysis to better understand the variability in LC outcomes across Ontario and define patient, disease, and system factors that contribute to regional differences*
**Methods**				
Study design	4	Present key elements of study design early in the paper	2–3	*2. Study Design and Methods*
Setting	5	Describe the setting, locations, and relevant dates, including periods of recruitment, exposure, follow-up, and data collection	3	*new diagnosis of LC between January 1st, 2007 and December 31st, 2017* *Maximum follow-up date was December 31st, 2019, at death, or end of OHIP eligibility*
Participants	6	(*a*) *Cohort study*—Give the eligibility criteria, and the sources and methods of selection of participants. Describe methods of follow-up *Case-control study*—Give the eligibility criteria, and the sources and methods of case ascertainment and control selection. Give the rationale for the choice of cases and controls *Cross-sectional study*—Give the eligibility criteria, and the sources and methods of selection of participants	2–3	Table 1, Figure 1 *2.1. Case Definitions, Demographics and Study Outcomes*
		(*b*) *Cohort study*—For matched studies, give matching criteria and number of exposed and unexposed *Case-control study*—For matched studies, give matching criteria and the number of controls per case		N/A
Variables	7	Clearly define all outcomes, exposures, predictors, potential confounders, and effect modifiers. Give diagnostic criteria, if applicable	3	*2.1. Case Definitions, Demographics and Study Outcomes*
Data sources/ measurement	8 *	For each variable of interest, give sources of data and details of methods of assessment (measurement). Describe comparability of assessment methods if there is more than one group	2	Table 1 *Cases were defined as patients over 18 years of age with a new diagnosis of LC between January 1st, 2007 and December 31st, 2017, by topography coding consistent with LC in OCR (C34) and exclusion of pathology not consistent with LC (e.g., hematopoietic malignancies, melanoma and Kaposi sarcoma), which is in keeping with Cancer Care Ontario’s definition. Additional patients were identified if LC was listed as cause or comorbidity on the death certificate, if not already captured in OCR data.*
Bias	9	Describe any efforts to address potential sources of bias	3	*cancer-specific 5-year survival for LC across LHINs, adjusted for patient age, sex, income quintile, rurality index, distance to nearest hospital (in kilometres), nearest hospital type (academic, community, small, unknown), LC stage at diagnosis, LC histology, co-morbidity status by ACG, timeliness of care (divided into 5 quintiles), and assessment by medical or radiation oncology. For this analysis, timeliness of treatment, radiation and medical oncology visits were modelled as time-varying covariates.*
Study size	10	Explain how the study size was arrived at	3–4	Figure 1
Quantitative variables	11	Explain how quantitative variables were handled in the analyses. If applicable, describe which groupings were chosen and why	3	Cox-Model
Statistical methods	12	(*a*) Describe all statistical methods, including those used to control for confounding	3	We used a Cox-model approach to compare overall and cancer-specific 5-year survival for LC across LHINs, adjusted for patient age, sex, income quintile, rurality index, distance to nearest hospital (in kilometers), nearest hospital type (academic, community, small, unknown), LC stage at diagnosis, LC histology, co-morbidity status by ACG, timeliness of care (divided into 5 quintiles), and assessment by medical or radiation oncology. For this analysis, timeliness of treatment, radiation and medical oncology visits were modelled as time-varying covariates. The Wald Chi-Square test was used to assess for statistical differences between groups.
		(*b*) Describe any methods used to examine subgroups and interactions		
		(*c*) Explain how missing data were addressed	3	Patients with missing data were excluded from relevant analysis. E.g. lung cancer survival estimate excluded patients with no prior OCR record and diagnosed at death as cancer was only identified at time of death
		(*d*) *Cohort study*—If applicable, explain how loss to follow-up was addressed *Case-control study*—If applicable, explain how matching of cases and controls was addressed *Cross-sectional study*—If applicable, describe analytical methods taking account of sampling strategy		
		(*e*) Describe any sensitivity analyses		N/A
**Results**				
Participants	13 *	(*a*) Report numbers of individuals at each stage of study—eg numbers potentially eligible, examined for eligibility, confirmed eligible, included in the study, completing follow-up, and analysed	4	Figure 1
		(*b*) Give reasons for non-participation at each stage		*N/A*
		(*c*) Consider use of a flow diagram		Figure 1
Descriptive data	14 *	(*a*) Give characteristics of study participants (eg demographic, clinical, social) and information on exposures and potential confounders	4-5	Table 2
		(*b*) Indicate number of participants with missing data for each variable of interest		N/A
		(*c*) *Cohort study*—Summarise follow-up time (eg, average and total amount)		
Outcome data	15 *	*Cohort study*—Report numbers of outcome events or summary measures over time	8-9	Table 4, Table 5
		*Case-control study*—Report numbers in each exposure category, or summary measures of exposure		
		*Cross-sectional study*—Report numbers of outcome events or summary measures		
Main results	16	(*a*) Give unadjusted estimates and, if applicable, confounder-adjusted estimates and their precision (eg, 95% confidence interval). Make clear which confounders were adjusted for and why they were included	8-9	Table 4, Table 5
		(*b*) Report category boundaries when continuous variables were categorized		
		(*c*) If relevant, consider translating estimates of relative risk into absolute risk for a meaningful time period		
Other analyses	17	Report other analyses done—eg analyses of subgroups and interactions, and sensitivity analyses		N/A
**Discussion**				
Key results	18	Summarise key results with reference to study objectives	11	*Significant variability in LC survival exists across health regions in Ontario. Our data demonstrate that both patient- and disease-related characteristics contribute significantly to this observed variability in survival, with LC stage at diagnosis and histologic subtype exhibiting the greatest impact on survival. However, variability in patient and disease characteristics between health regions did not account for all the observed variability in adjusted cancer-specific survival across LHINs, suggesting that other system factors may play a contributing role. We adjusted for several system factors in a multivariate analysis including timeliness of care, size of closest hospital, and specialist consultation, yet variability in survival across health regions persisted.*
Limitations	19	Discuss limitations of the study, taking into account sources of potential bias or imprecision. Discuss both direction and magnitude of any potential bias	13	*One limitation of our study was the inability to obtain LHIN level smoking rates or patient access to smoking cessation counselling.* *Another limitation is related to the imperfect nature of database cohort analyses, recognizing that coding of information is not always accurate.* *Our cohort definition was in keeping with Cancer Care Ontario’s definition, in an effort to minimize inaccuracies*.
Interpretation	20	Give a cautious overall interpretation of results considering objectives, limitations, multiplicity of analyses, results from similar studies, and other relevant evidence	14	*Significant variability in LC survival exists across health regions in Ontario, not completely accounted for by variability in those patient and disease characteristics available in our data, suggesting that other patient or system factors play a contributing role. A regional approach to improvement efforts will be required to identify the root causes contributing to unique regional system factors that influence survival to develop targeted improvement strategies. Governing bodies should consider the uniqueness of individual health regions to ensure that resources are equitably distributed in such a way that address unique regional needs and should consider tracking additional quality metrics, beyond timeliness of care, that have the most potential to impact LC survival.*
Generalisability	21	Discuss the generalisability (external validity) of the study results	14	*Finally, since there is no reason to believe that these variations across regions is unique to the province of Ontario, our findings are likely relevant to other jurisdictions with similar health care systems in Canada and elsewhere.*
**Other information**				
Funding	22	Give the source of funding and the role of the funders for the present study and, if applicable, for the original study on which the present article is based	14	*Funding: William M. Spear Endowment Fund in Pulmonary Research and the Richard K. Start Memorial Fund, Queen’s University, Ontario, Canada. This study was supported by ICES, which is funded by an annual grant from the Ontario Ministry of Health and Long-Term Care (MOHLTC). Parts of this material are based on data and information compiled and provided by CIHI and Ontario Health. The analyses, conclusions, opinions and statements expressed herein are solely those of the authors and do not reflect those of the funding or data sources; no endorsement is intended or should be inferred*

* Give information separately for cases and controls in case-control studies and, if applicable, for exposed and unexposed groups in cohort and cross-sectional studies. Note: An Explanation and Elaboration article discusses each checklist item and gives methodological background and published examples of transparent reporting. The STROBE checklist is best used in conjunction with this article (freely available on the Web sites of PLoS Medicine at http://www.plosmedicine.org/ (accessed on 3 February 2022), Annals of Internal Medicine at http://www.annals.org/ (accessed on 3 February 2022), and Epidemiology at http://www.epidem.com/, (accessed on 3 February 2022)). Information on the STROBE Initiative is available at www.strobe-statement.org (accessed on 3 February 2022).
